# CGMformer: a novel deep-learning model promising for early detection of prediabetes to effectively prevent type 2 diabetes

**DOI:** 10.1093/nsr/nwaf188

**Published:** 2025-05-14

**Authors:** Xiao-ding Peng

**Affiliations:** Department of Biochemistry and Molecular Genetics, College of Medicine, University of Illinois at Chicago, USA

Type 2 diabetes (T2D) is a chronic disorder in glucose metabolic homeostasis. Its crucial molecular pathological mechanism is driven by both genetic and environmental factors, leading to obstruction of the insulin signal transduction pathway, resulting in characteristic insulin resistance and β-cell dysfunction [1–3]. Despite ongoing efforts in lifestyle intervention, the global incidence of T2D continues to rise annually [4]. To seize the time window of the reversible stage of this disorder and to effectively reduce its incidence, early detection is an important starting step. Therefore, it is imperative to have a relatively accurate yet convenient measure to detect early-stage prediabetes. A recent interdisciplinary study—conducted by a mathematics team led by Yong Wang and Luonan Chen, and a medical team led by Weiping Jia and Huating Li—developed a deep-learning model named CGMformer, meeting such a demand [5]. CGMformer was pretrained on a large-scale and diverse corpus of Continuous Glucose Monitoring (CGM) data from the Nationwide Multicenter CGM data sets and unlabeled National Real-World CGM data. CGM is used for monitoring blood glucose on a continual basis with a device consisting of a small electrode probe sensor placed under the skin, an attached transmitter, and a separate receiver that displays glucose levels.

**Figure 1.**
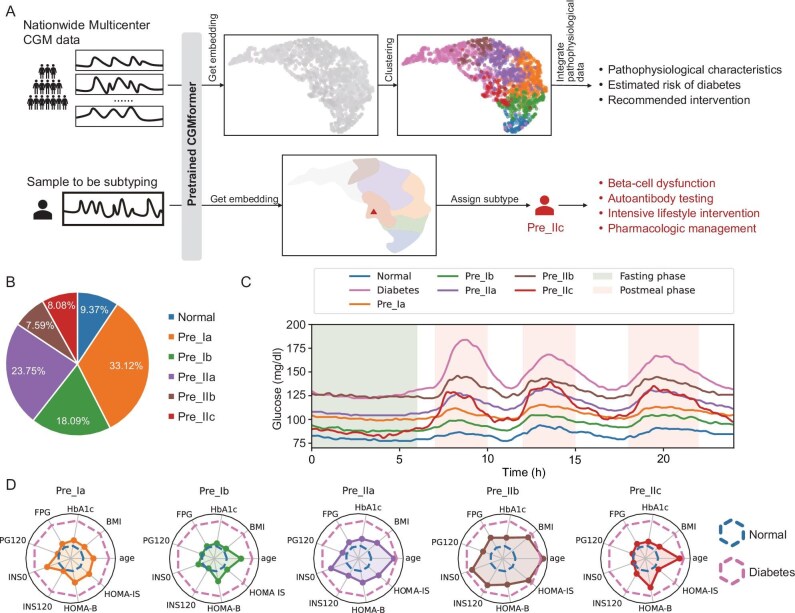
CGMformer enables detailed and comprehensive subtyping for non-diabetes. (A) Framework for construction and subtyping with CGMformer_type. (B) Ratio of each subtype in the Nationwide Multicenter CGM data. (C) Median CGM profile for subtypes, offering insights into the unique glucose dynamics of different subtypes. (D) Clinical characteristics for each subtype. FPG: fasting plasma glucose, PG120: post-meal 120-minute glucose, INS0: fasting plasma insulin, INS120: post-meal 120-minute insulin, HOMA-B: homeostatic model assessment of β cell function, HOMA-IS: homeostasis model assessment for insulin sensitivity, BMI: body mass index. Figure adapted from ref. [5] with the assistance of Yong Wang and Yurun Lu.

CGMformer not only employed CGM data but also integrated these data with an individual's clinical or lifestyle information across individuals and within individuals and captured complex glucose dynamics. CGMformer enabled several clinical applications, among which non-diabetes subtyping (CGMformer_type) is quite attractive (Fig. 1A–C). CGMformer_type, in early detection of the potential onset of T2D, outperformed other methods currently used, such as glucose level testing on static time points, or hemoglobin A1c (HbA1c).

CGMformer_type subtyped individuals of non-diabetes into Normal, Pre_Ia, Pre_Ib, Pre_IIa, Pre_IIb and Pre_IIc (Fig. 1B). The majority of Pre_Ia and Pre_Ib were clinically diagnosed as having normal glucose tolerance (NGT), but CGMformer, demonstrating its early warning advantage, flagged the two subtypes with potential risk of prediabetes. For subtypes Pre_IIa and Pre_IIb, which are already of certain prediabetes characteristics, besides providing personalized dietary recommendations CGMformer also recommends metformin intervention for some individuals of Pre_IIb. This echoes a suggestion that metformin should be administered as early as the prediabetes stage in addition to lifestyle intervention to enhance the possibility of reversing prediabetes [2,6]. Pre_IIc is a unique subtype with high variance (Fig. 1C). It lacks insulin resistance but displays β-cell dysfunction and insulin deficiency (Fig. 1D). Further differential diagnosis would be necessary to rule out the possibility that this subtype is actually a type 1 diabetes (T1D) subtype called latent autoimmune diabetes in adults (LADA). The insulin deficiency of LADA is caused by an autoimmune attack on islet β cells [7] while β-cell dysfunction of T2D is mainly due to insulin resistance at molecular levels within the β-cell itself [1,3]. LADA can be identified by autoantibody testing. The incidence of LADA misdiagnosed as T2D in China was reported to be 8.62% in a multicenter study [7]. The proportion of Pre_IIc was 8.08% by CGMformer_type (Fig. 1B). If we can assume, pending future validation of autoantibody testing, that the majority of Pre_IIc is LADA, the 8.08% proportion would be justified in that this proportion was generated from a larger scope of participants comprising not only suspected prediabetes (including those still manifesting NGT) but also the normal subtype. This could imply that CGMformer_type can detect LADA even at a very early stage which glucose levels testing at static time points cannot detect. This would further support CGMformer's sensitivity and reliability.

Apart from CGMformer_type for early detection of prediabetes, CGMformer demonstrated advantages in several other clinical applications. These include CGMformer_C, which shows superior performance in predicting the risk of diabetic complications; CGMformer_Diet, which predicts postprandial glucose and provides personalized dietary recommendations; and more [5]. With these advantages, and with further refinement to enhance its generalizability and reliability—such as incorporating a more diverse range of samples from different ethnicities and geographical regions for validation, and comparing model performance across various populations—CGMformer could be more robustly positioned for global dissemination. We foresee that its scope of applicability will be further expanded, including promoting the application of CGM in much earlier detection of prediabetes, and supporting future studies on the early pharmaceutical intervention of prediabetes, which is a far more important step to reduce the incidence of T2D.
